# Cbl downregulation increases RBP4 expression in adipocytes of female mice

**DOI:** 10.1530/JOE-17-0359

**Published:** 2017-11-07

**Authors:** Gulizar Issa Ameen, Silvia Mora

**Affiliations:** Department of Cellular and Molecular PhysiologyInstitute of Translational Medicine, University of Liverpool, Liverpool, UK

**Keywords:** RBP4, adipokine, Cbl, adipocyte, insulin

## Abstract

Obesity leads to adipose tissue dysfunction, insulin resistance and diabetes. Adipose tissue produces adipokines that contribute to regulate insulin sensitivity. In turn, insulin stimulates the production and release of some adipokines. Casitas-b-lymphoma proteins (c-Cbl, Cbl-b and Cbl3) are intracellular adaptor signalling proteins that are rapidly phosphorylated by activation of tyrosine kinase receptors. c-Cbl is rapidly phosphorylated by insulin in adipocytes. Here, we tested the hypothesis that Cbl signalling regulates adipokine expression in adipose tissue. We determined the adipokine profile of WAT of Cbl−/− and Cbl+/+ mice in the C57BL6 background. Female Cbl−/− mice exhibited altered expression of adiponectin, leptin and RBP4 in visceral adipose tissue, while no significant changes were seen in male mice. TNFα and IL6 levels were unaffected by Cbl depletion. RBP4 expression was unchanged in liver. Adipose tissue of Cbl−/− animals showed increased basal activation of extracellular regulated kinases (ERK1/2) compared to Cbl+/+. c-Cbl knockdown in 3T3L1 adipocytes also increased basal ERK phosphorylation and RBP4 expression. Inhibition of ERK1/2 phosphorylation in Cbl-depleted 3T3L1 adipocytes or in adipose tissue explants of Cbl−/− mice reduced RBP4 mRNA. 17β-Estradiol increased RBP4 mRNA in adipocytes. Cbl depletion did not change ER expression but increased phosphorylation of ERα at S118, a target site for ERK1/2. ERK1/2 inhibition reduced phosphoER and RBP4 levels. These findings suggest that Cbl contributes to regulate RBP4 expression in adipose of female mice through ERK1/2-mediated activation of ERα. Since Cbl signalling is compromised in diabetes, these data highlight a novel mechanism that upregulates RBP4 locally.

## Introduction

Casitas b-lymphoma (Cbl) is a proximal insulin receptor adaptor protein identified as necessary for insulin-mediated activation of glucose transport in adipocytes (Baumann *et al*. 2000, Chiang *et al*. 2001, Chang *et al*. 2002, Liu *et al*. 2002, 2003). Three Cbl isoforms are present in mammalian cells (c-Cbl, Cbl-b and Cbl3). Cbl3 is a truncated isoform present only in the skin, while c-Cbl and Cbl-b are quite ubiquitous and become rapidly phosphorylated on tyrosine residues in response to insulin and other growth factors. c-Cbl is the predominant isoform expressed in mature adipocytes (Liu *et al*. 2003). We reported that C-Cbl (hereafter Cbl) is also quickly phosphorylated by insulin *in vivo* in heart and skeletal muscle albeit in these tissues it appears to regulate other intracellular proteins (Gupte & Mora 2006). Cbl phosphorylation and expression is compromised in diabetic animal models (Gupte & Mora 2006). In addition, Cbl proteins also contain a RING finger domain that allows them to function as E3-ubiquitin ligase enzymes and thus facilitate protein degradation. Whole-body disruption of c-Cbl gene in mice in the Jvs129 background resulted in reduced fat peripheral stores and increased fatty acid oxidation in skeletal muscle and whole-body insulin sensitivity (Molero *et al*. 2004). These mice have also exhibited resistance to the deleterious effects of a high-fat diet (Molero *et al*. 2006).

White adipose tissue (WAT) has an important role in maintaining glucose and lipid homeostasis and thus in maintaining health. WAT is a dynamic tissue that stores surplus energy in the form of triglycerides, but it also produces a plethora of bioactive molecules collectively called adipokines that help regulate biological functions in other cells and organs. Some of these proteins like adiponectin increase insulin sensitivity in peripheral organs, some arepro-inflammatory such as IL6 or TNFa and inhibit insulin action and others like leptin can function both ways.

Adiponectin (also called ACRP30) is an adipokine with insulin-sensitizing functions in liver and skeletal muscle (Berg *et al*. 2002, Yamanuchi *et al*. 2002, Kadowaki *et al*. 2006), as well as anti-inflammatory (Takemura *et al*. 2007, Ohashi *et al*. 2010, Mandal *et al*. 2011) and anti-atherogenic effects (Ouchi *et al*. 2001, Okamoto *et al*. 2002, 2008, Lovren *et al*. 2010). Adiponectin circulating levels are inversely correlated with BMI, visceral adiposity and insulin resistance (Arita *et al*. 1999, Pajvani & Scherer 2003, Bouatia-Naji *et al*. 2006) (for a recent reviews Ouchi *et al*. 2011, Kwon & Pessin 2013, Ohashi *et al*. 2014, 2015).

Leptin is a small WAT adipokine that regulates feeding behaviour through the hypothalamic regulation in central nervous system (Zhang *et al*. 1994). Leptin plasma levels are elevated in obese rodents and humans due to increased adipose tissue content and leptin resistance (Friedman & Halaas 1998). Leptin stimulates monocytes and macrophages to produce proinflammatory cytokines including: IL6, TNFa and IL12 (Gainsford *et al*. 1996) and stimulates CCL2 production in human hepatic stellate cells (Aleffi *et al*. 2005).

Retinol-binding protein 4 (RBP4) is both an adipokine and liver-derived protein that transports retinol (vitamin A) in the blood (Tamori *et al*. 2006). It has been implicated in the metabolic syndrome and adipose tissue inflammation (Kovacs *et al*. 2007, Boyraz *et al*. 2013). RBP4 expression in adipose tissue correlates positively with inflammation (Yao-Borengasser *et al*. 2007), expression of proinflammatory cytokines and adiposity (Klöting *et al*. 2007) and inversely with GLUT4 protein levels (Yang *et al*. 2005). Recombinant RBP4 administration induced insulin resistance in mice (Yang *et al*. 2005). RBP4 levels are increased in obese and insulin-resistant humans (Graham *et al*. 2006) and mouse models. Genetic (Moraes-Vieira *et al*. 2014) or pharmacologic elevation of serum RBP4 causes insulin resistance and hepatic steatosis in mice (Lee *et al*. 2016). Recent studies in transgenic mice models overexpressing *Rbp4* gene in adipose tissue have revealed that RBP4 causes inflammation in adipose tissues by activating macrophages independently of its retinol-binding status and via activation of the Toll-like 4 receptor (Norseen *et al*. 2012), as well as activating innate immunity (Moraes-Vieira *et al*. 2014, 2016).

Here, we tested the hypothesis that Cbl signalling in adipose tissue may regulate adipokine expression. We examined the expression and circulating levels of several adipokines involved in insulin sensitivity in the c-Cbl null animal model and their WT littermates and their expression in c-Cbl knocked-down 3T3L1 adipose cells. We report that Cbl depletion in adipocytes increases basal activation of extracellular regulated kinases (ERK1/2) and results in enhanced expression of RBP4 in adipocytes of female mice via activation of the oestrogen receptor.

## Materials and methods

### Reagents plasmids and antibodies

Tissue culture reagents (FBS, trypsin, penicillin/streptomycin, insulin, IBMX, dexamethasone, puromycin, 17-β estradiol) were from Sigma-Aldrich. MEK1/2 inhibitors PD98059 and U0126 were obtained from Sigma-Aldrich. Adipokine ELISA kits for RBP4 (cat. Number DY3476, assay range: 93.8–6000 pg/mL), adiponectin (cat. Number DY1119, assay sensitivity 31–2000 pg/mL) and leptin (cat. Number DY498, assay range 125–8000 pg/mL) were from R&D Systems (USA), IL6 (cat. Numbers capture: detection: from and TNFa (cat. number from BD Biosciences) and ELISA antibodies were from ThermoFisher Scientific and used at 2 µg/mL). The PKLO.1 puro empty plasmid (cat. number SHbib1), the PKLO.1 plasmid carrying a non-targeting shRNA (NT-shRNA) sequence (cat. SHbib2) and PKLO.1 puro vectors carrying shRNAs for c-CBL proteins were from Sigma (MISSION shRNA). The PMDG2 and PMCV-dR8.74 vectors to generate the lentiviral vectors were a gift from Dr Antonio Zorzano (IRB, Barcelona, Spain). Antibodies: anti-IRS1 (cat. number 2382, 1:1000), phospho-AKT (Ser473) (cat. number 9271, 1:1000), total AKT (cat. number 2920, 1:1000), phosphoERK1/2 (T202/T204) (cat. number 910, 1:1000), total ERK1/2 (p44/p42) (cat. number 9107, 1:1000) and phosphoER (S118) (cat. number 2511, 1:1000) were from Cell Signalling. Total ERa antibody was from Abcam (cat. number ab108398, 1:2000), anti-β-tubulin (catalogue number T4026, 1:2000) and antiACTIN (catalogue number A2066, 1:2000) antibodies were from Sigma-Aldrich, and anti-GLUT4 antibody was a gift from Dr Jeffrey Pessin (Albert Einstein, NY, USA).

### Animals, genotyping and diet intervention

c-Cbl-null mice on C57BL6/J background were obtained from the National Institute of Health. C57BL6/J wild-type controls (WT) were purchased from the Jackson Laboratories. Cbl-null and WT animals were bred to obtain heterozygous animals (Cbl^+/−^) and the colony expanded through het × het crossings to obtain experimental cohort of animals. In all experiments, age and gender WT littermate control animals from our colony were used. For some experiments in addition to het × het crossings, Cbl^−/−^ × Cbl^−/−^ and Cbl^+/+^ × Cbl^+/+^ pairs from our colony were established to assure enough animals per each genotype were available. Genotypes were determined by PCR of genomic DNA using the following primer sets: LOXP: 5′ TGG CTG GAC GTA AAC TCC TCT TCA GAC CTA ATA AC 3′; CBL-10: 5′ GAC GAT AGT CCC GTG GAA GAG CTT TCG ACA 3′; CBL-11: 5′ CCT AAG TGG TAG GAT TAT AAT TGC AAG CCA CCA C 3′ and CBL-13: 5′ TCC CCT CCC CTT CCC ATG TTT TTA ATA GAC TC 3′, which amplify the targeted and non-targeted genes, respectively. Animals were housed at 12-h light/darkness cycle and fed *ad libitum* with standard chow diet. Weight of animals was monitored weekly. All procedures were carried out in accordance with the UK Animal (Scientific Procedures) Act 1986 and Home Office licenses

Glucose and insulin tolerance tests were carried out as we previously reported (Yang *et al*. 2001) at week 12.

### Cells

3T3L1 cells were obtained from the ATCC. Culture and differentiation of cells was carried out as we described previously (Xie *et al*. 2006) in cell passages <p10. Generation of 3T3L1 cell lines stably expressing shRNAs for c-Cbl or empty PKLO puro vector or NT-shRNA were carried out as we described previously (Carson *et al*. 2013).

Culturing of adipose tissue explants was carried out as described (Fried & Moustaid-Moussa 2001). Briefly, WAT was dissected and fragmented into small (1–2 mm) pieces under sterile conditions, and subsequently incubated for 2 h in Dulbecco Modified Eagle’s media (DMEM). Following the first 2 h of incubation, the media was changed to fresh DMEM and cultured further for up to 24 h. At that time, insulin was added for 30 min at final concentrations of 0, 1, 10 and 100 nM insulin (or as indicated in figure legends); tissues were then snap frozen in liquid nitrogen and stored at −80°C until analysis.

### Immunohistochemistry of adipose tissue

Dissected perigonadal WAT was fixed in 4% paraformaldehyde/PBS overnight at 4°C, rinsed in PBS, dehydrated through a series of descending graded alcohols and embedded in paraffin. Tissue was sectioned in a microtome at 5–7 µm thick sections, dewaxed in histoclear (5 min, twice), rehydrated stained with Mayer’s haematoxylin (8 min), rinsed in water and acidified water (10 s). Sections were counterstained with eosin (2 min) washed in tap water, dehydrated in ascending graded alcohols, cleared in xylene and mounted using DPX mounting medium. Sections were visualized in a Leica inverted microscope at 40× magnification and pictures were taken with colour camera. Image analysis of histological tissue preparations (cell size and diameter) were carried out using ImageJ software (NIH).

### Tissue/cell lysate preparation and immunoblotting

Tissue homogenates and whole-cell lysates were obtained as previously described (Mora *et al*. 2001). Briefly, tissue samples were homogenised in a DOUNCE glass homogenizer, in ice-cold lysis buffer (NaCl 100 mM; EDTA 1 mM; 1% Triton x-100; NaF 50 mM; 2 mM sodium pyrophosphate; 1 mM sodium vanadate; 1 mM phenylmethylsulfonyl fluoride; 2 µg/mL aprotinin; 2 µg/mL pepstatin A and 2 µg/mL leupeptin). Samples were incubated at 4°C for 30 min and centrifuged 15,300 ***g*** for 15 min at 4°C. Protein concentration of the supernatant was determined using the Bio-Rad Protein Assay Kit. Samples were separated on a SDS-PAGE, transferred to nitrocellulose membranes, blotted in 5% non-fat milk in Tris-buffered saline (pH 7.6) and subsequently immunoblotted with primary antibodies and fluorescent-labelled secondary antibodies IRDye 800 cw (cat. number 92632210 at 1:15,000) and IRDye 680RD (cat. number 926-68071 at 1:20,000) (LICOR). Membranes were washed in Tris-buffered saline containing 0.1% Tween and visualized in a LI-COR Odyssey system. Quantification of blots relative to reference protein as indicated in the figure legends was carried out using ImageJ (NIH).

### ELISA determination of adipokine content

Adipose tissue or 3T3L1 cells were obtained in lysis buffer by homogenization as described earlier and as we reported previously (Mora *et al*. 2001). Expression of adipokines (RBP4, adiponectin, leptin, TNFa and IL6) were determined by ELISA using commercially available kits and antibodies as we have previously reported (Xie *et al*. 2008, Carson *et al*. 2013). Adipokine content was determined in a 10 µL aliquot of lysate using standards supplied by the manufacturer, and the results were normalized to the total protein content in the lysate as determined by Bradford. Plasma from tail bleeds was collected in EDTA-coated tubes. The sample was centrifuged 10 min at 400 ***g*** at room temperature and the supernatant containing the plasma was transferred to a new tube and frozen at −80°C until used. For ELISA, a 10 µL aliquot was used. A standard curve with recombinant protein provided by the kit was used in each assay, and when necessary, the plasma was diluted in PBS so that the adipokine values were within the standard curve.

### Total RNA extraction and qPCR

Total RNA was isolated using Tri reagent (Sigma-Aldrich) following the manufacturer’s instructions. RNA was quantitated by spectrophotometry and visualized in an agarose gel. Total RNA was reverse transcribed to cDNA using an iScript cDNA synthesis kit (BIO-RAD) following the manufacturer’s instructions. Validated TaqMan probes for Rbp4 and 18S (assay IDs: Mn00803264-31 and Hs 99999901, respectively) were obtained from Life Technologies. The resulting cDNA was amplified using iTAQ probes and iSCript qPCR kit (Life Technologies). Oestrogen receptor isoforms α and β (accession numbers NM007956.5 and BC145329.1, respectively) were amplified using Kapa Sybr green Fast mix from Roche and the following primers: ERα: F: 5′TGATTGGTCTCGTCTGGCG3′; R: 5′CATGCCCTCTACACATTTACC3′; ERβ: F: 5′CTGGCTAACCTCCTGATGCT3′; R: 5′CCACATTTTTGCACTTCATGTTG3′. The primers produce amplicons of 100 bp and 91 bp, respectively. The conditions of the reaction were denaturation 95°C 30 s, annealing 60°C 20 s, extension 72°C 30 s, for 40 cycles. A melting curve was run at the end of each run. Relative quantification was carried out using the ΔΔCt method using 18S gene expression (primer sequences: forward: 5′TCAAGAACGAAAGTCGGAGG 3′ and reverse: 5′GGACATCTAAGGGCATCACA3′: for normalization, as we have previously reported (Yang *et al*. 2012).

### Statistical analysis

Statistical analyses were carried out using GraphPad Prism 6 software (GraphPad software). Student *t*-tests or analysis of variance (ANOVA) analyses with Sidak’s *post hoc* test were carried out as indicated in the figure legends, with a confidence interval of 95% and statistical significance was considered if *P* < 0.05.

## Results

### c-Cbl depletion increases whole body glucose sensitivity in male mice and alters adipokine expression in WAT of female mice

**Figure 1:**
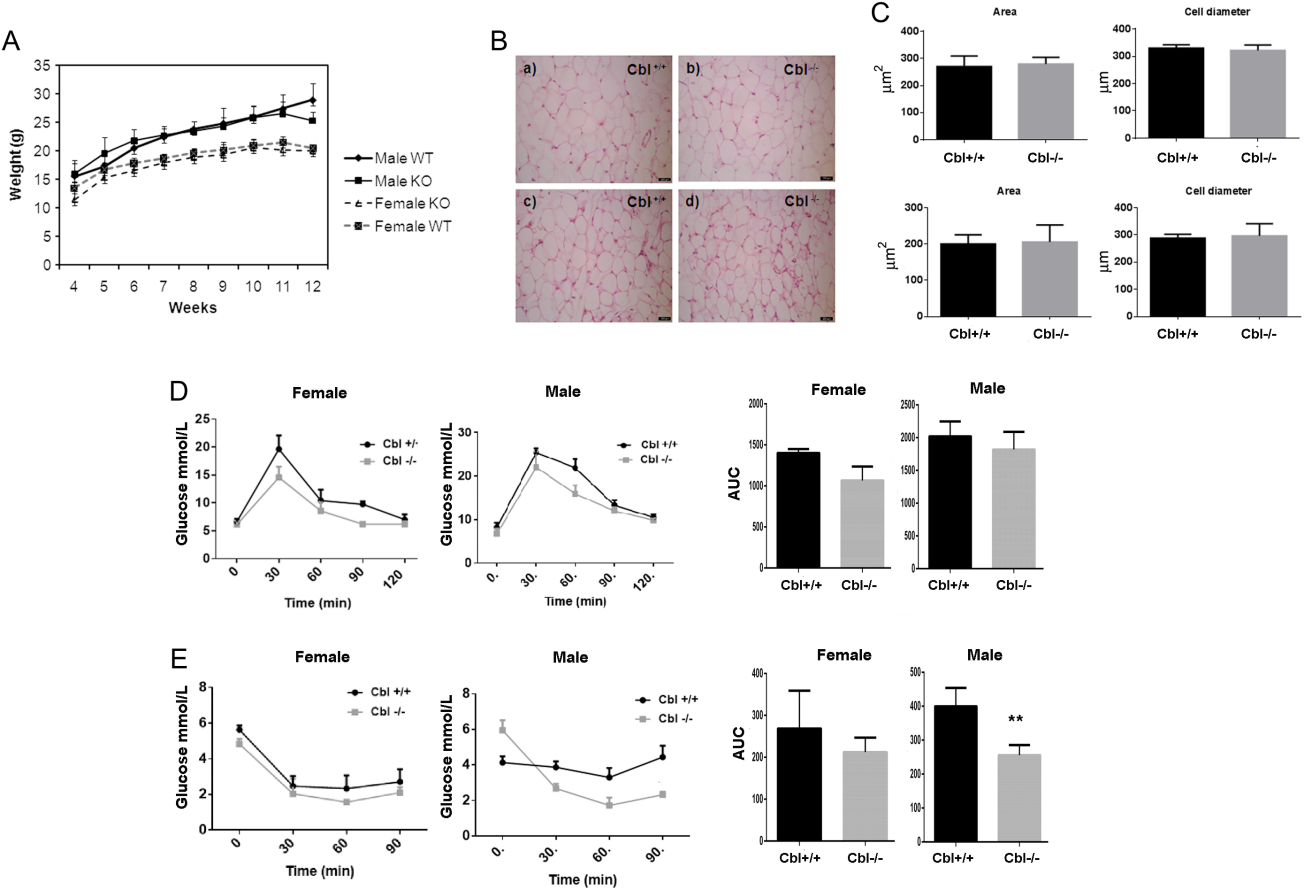
(A) Growth curve of WT and CBL-null mice. Data show mean ± s.e.m. of weekly weight. Cbl^+/+^: male *n* = 18, female *n* = 12; Cbl^−/−^: male *n* = 14, female *n* = 17. Statistical analysis multiple *t*-test, not significant. (B) Haematoxylin–eosin staining of perigonadal white adipose tissue of Cbl^+/+^ and Cbl^−/−^ mice. Representative images shown of pictures obtained at 40× magnification of Cbl^+/+^ *n* = 9 and Cbl^−/−^ *n* = 12 mice. Panels a and b are male and panels c and d are female mice. (C) Quantification: ImageJ was used to quantify cell diameter of *n* = 100 cells and cell area *n* = 50 cells per genotype and gender. Top graphs correspond to male and bottom to female mice. Graphs show mean ± s.e.m. Statistical analysis: paired *t*-test, not significant. (D) Glucose tolerance test (GTT) and (E) insulin tolerance test (ITT) were carried out at 14 weeks. Mice were injected with glucose 2 g/kg of body weight (GTT) or insulin 0.75 U/kg of body weight (ITT) and at the times indicated, blood tail samples were obtained and glucose measured using a glucose meter. Graph show mean + s.e.m. of values. Male mice *n* = 4, female mice *n* = 3. Statistical analysis: paired *t*-test. Area under the curve (AUC) *P* values: female: GTT: *P* = 0.079, ITT: *P* = 0.442; male: GTT: *P* = 0.428, ITT: *P* = 0.0035.

c-Cbl-null mice on a Jvs129 background were shown to have reduced adiposity and to be more insulin sensitive than WT mice, even on a high-fat diet (Molero *et al*. 2004, 2006). Because the c-Cbl^−/−^ used in our studies was generated in a C57BL6 background, we first sought to determine growth, adiposity and *in vivo* whole-body glucose tolerance and insulin sensitivity of our mice. As shown in Fig. 1A, female mice were slightly smaller than male mice, but there were no differences in the weight of c-Cbl^−/−^ mice compared to c-Cbl^+/+^. We did not detect any significant differences in adipose tissue morphology, as revealed by eosin and hematoxylin staining of visceral adipose depots and adipocyte cell size quantification (Fig. 1B and C). Similarly to what was reported previously (Molero *et al*. 2004), the null mice showed a tendency towards an increase in glucose tolerance, although it did not reach statistical significance for neither gender (Fig. 1D). Male mice, however, showed improved insulin sensitivity (Fig. 1E), whereas this was not observed in female mice.

**Figure 2:**
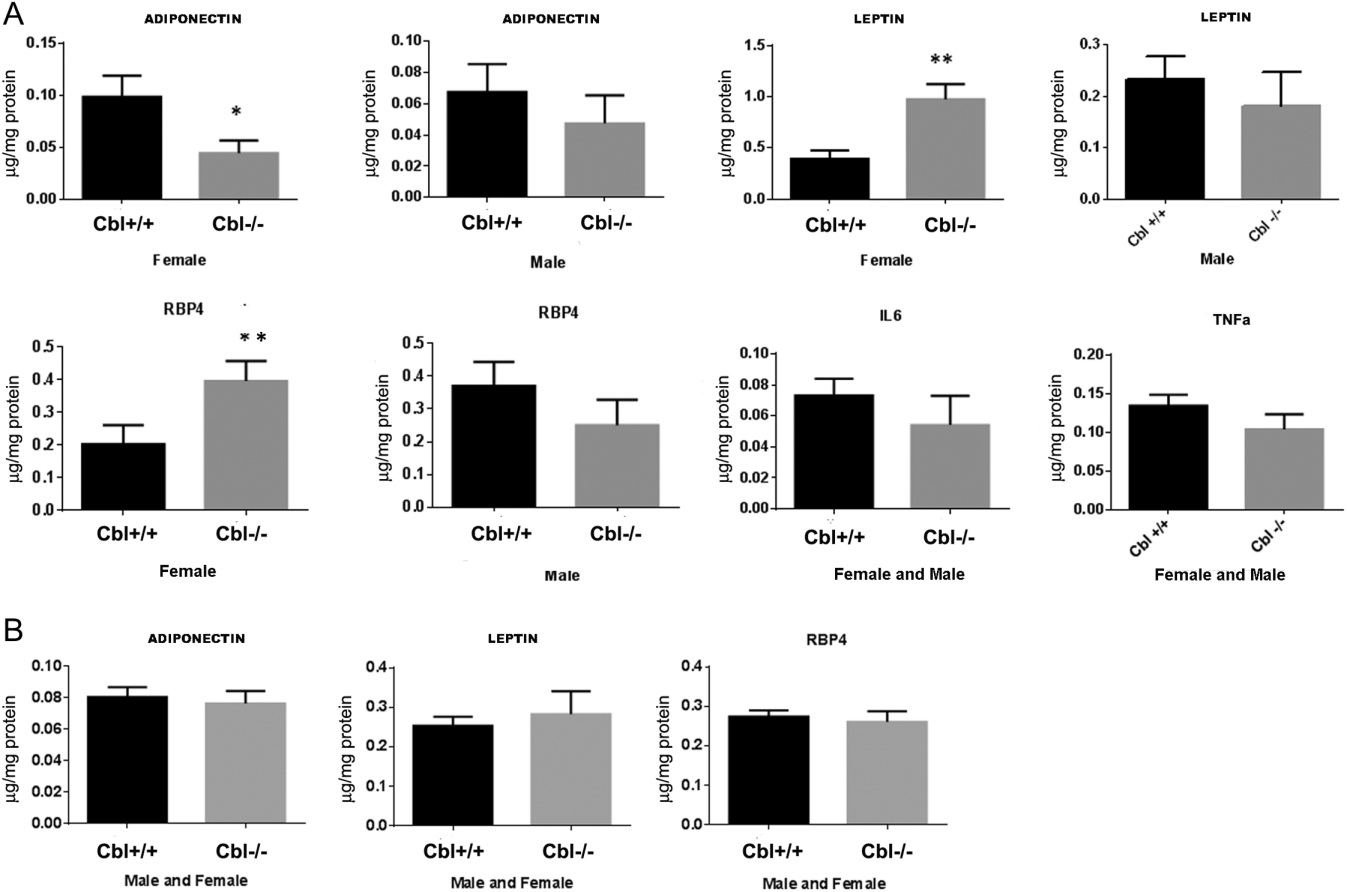
Cbl^−/−^ mice shows altered expression of adipokines in WAT of female mice. (A) Tissue lysates were obtained from perigonadal WAT and adipokines quantified by ELISA as specified in the Methods section. Adipokine levels were normalized by the total protein content in the lysate. Graphs show mean ± s.e.m. for adiponectin, leptin and RBP4 data from Cbl^+/+^: *n* = 15 males and *n* = 13 female mice; Cbl^−/−^: *n* = 6 males and *n* = 12 females. For IL6 and TNFa Cbl^−/−^ *n* = 4 and cbl^+/+^ *n* = 10 mice. Statistical analysis: paired *t*-test. * indicates *P* < 0.05; ** indicates *P* < 0.01. (B) Plasma levels of adipokines. Mice were fasted for 6 h and plasma was drawn from the tail. Plasma adipokines were quantitated by ELISA. Graphs show mean ± s.e.m. of *n* = 10 Cbl^+/+^ and *n* = 5 Cbl^−/−^. Statistical analysis paired *t*-test. Non-significant.

We next determined the adipokine profile expression in visceral adipose tissue and plasma by ELISA. In the steady state, there was a marked gender dimorphism in the content of some adipokines in WAT. In adipose tissue of female mice, adiponectin levels were decreased, whereas leptin and RBP4 levels were increased. No significant changes were observed in male Cbl^−/−^ mice compared to Cbl^+/+^ mice. TNFα or IL6 in WAT was not significantly different to that obtained in the WT mice for either gender (Fig. 2A). Circulating plasma levels for adiponectin, leptin or RBP4 were unremarkable in the Cbl-null animals compared to WT mice (Fig. 2B).

**Figure 3:**
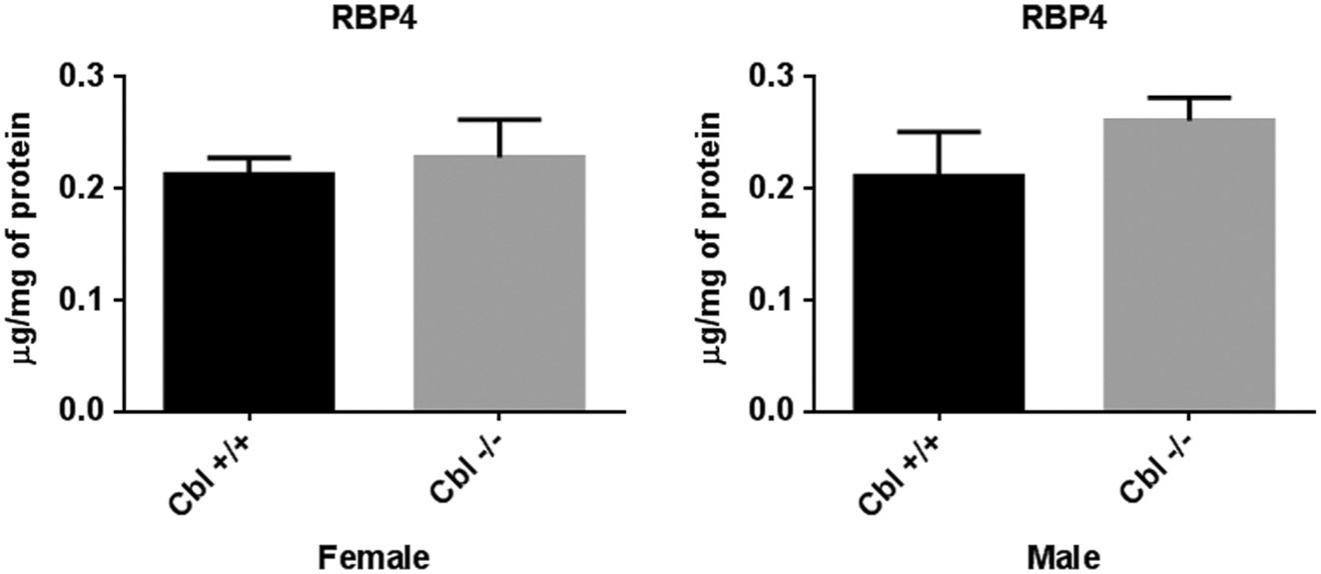
RBP4 expression in liver. Liver tissue lysates were obtained and RBP4 quantified by ELISA. Graphs show mean ± s.e.m. of RBP4 normalized to total protein. Cbl^+/+^: male *n* = 4, female *n* = 5; Cbl^−/−^: male *n* = 4, female *n* = 8. Paired *t*-test, * indicates *P* < 0.05.

Despite the changes in RBP4 protein levels in WAT, no significant changes were detected in the liver of Cbl^−/−^ mice compared to Cbl^+/+^ animals (Fig. 3).

### Depletion of c-Cbl increases ERK1/2 activation in WAT of mice and in cultured 3T3L1 adipose cells

**Figure 4:**
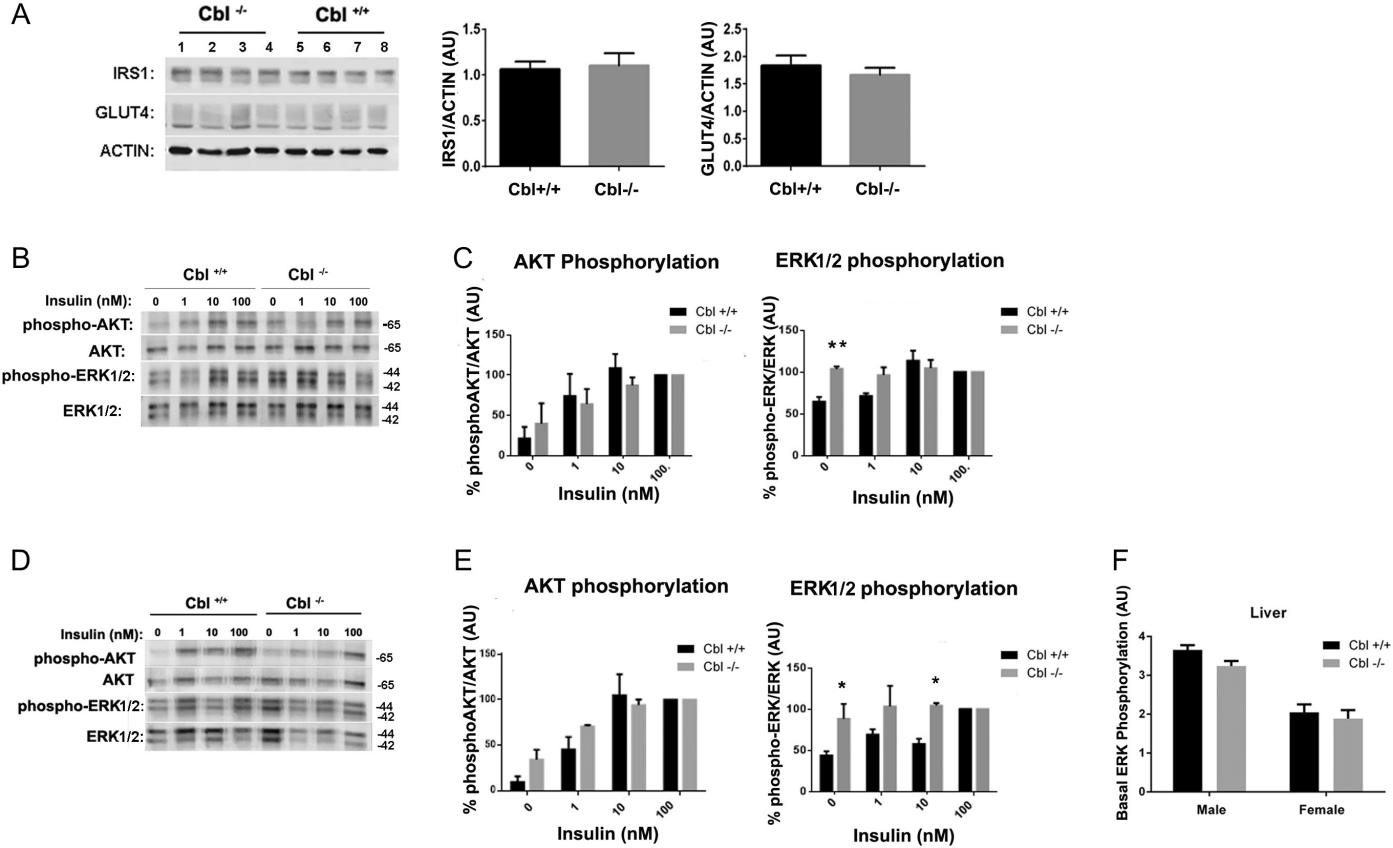
Increased activation of ERK in adipose tissue of c-Cbl^−/−^. (A) White adipose tissue extracts of c-Cbl^+/+^ and c-Cbl^−/−^ mice were immunoblotted for IRS1 and GLUT4, quantification vs actin is shown on the right panel. (B, C and D) White adipose tissue explants from c-Cbl^+/+^ and c-Cbl^−/−^ mice were obtained and either left untreated or treated with insulin at the indicated concentrations (0–100 nM). Tissue extracts were obtained, loaded onto SDS-PAGE and immunoblotted with antibodies as indicated. Panel B shows data from male mice and (D) female mice. Panels (C) and (E) show quantifications of AKT and ERK1/2 phosphorylation relative to the total AKT or ERK1/2 protein respectively from 5 experiments. Graph shows mean ± s.e.m. of phosphorylation over basal as % of Ser473 (AKT) or ERK T202/204 and Thr185/Tyr187, respectively. Statistical analysis Two way ANOVA with Sidak’s test, * indicates *P* < 0.05; ** indicates *P* < 0.01. (F) Liver extracts were obtained from c-Cbl^+/+^ and c-Cbl^−/−^ mice (*n* = 4 per group) and immunoblotted for phosphoERK1/2 and tubulin. Graph shows mean ± s.e.m. of the quantification over tubulin (arbitrary units). Statistical analysis: *t*-test, not significant.

In order to determine the molecular mechanisms that may be involved in the upregulation of RBP4 expression in adipose tissue, we examined the insulin signalling pathways in the adipose tissue of c-Cbl-null mice and WT animals. Expression of the insulin-responsive substrate 1 (IRS1) or the glucose transporter GLUT4 was not altered in visceral WAT of null mice compared to controls (Fig. 4A and B). Perigonadal visceral adipose tissue explants were obtained from Cbl^−/−^ or Cbl^+/+^ animals and were incubated in the absence or presence of insulin at concentrations ranging from 0 to 100 nM. We then determined the activation of the phosphatidylinositol 3-kinase and ERK1/2 signalling pathways by monitoring the phosphorylation levels of the downstream serine threonine kinase AKT at Ser473 and the phosphorylation levels of p44/p42 ERK1/2 at Thr202/204. Insulin effectively activated phosphatidylinositol 3 kinase and AKT to a similar extent in both c-Cbl-null and WT mice (Fig. 4, male animals panels B, C; female animals panels D, E). However, while activation of ERK1/2 proteins was achieved to a similar extent with submaximal insulin concentrations in the two genotypes, the c-Cbl-null mice displayed a greater ERK1/2 phosphorylation in the basal (untreated) state, with approximately 40% more phosphorylation of p44/p42 compared to the WT (Fig. 4 panels C, E). Interestingly, this increase in ERK1/2 phosphorylation was not seen in the liver (Fig. 4F).

To confirm that the *Cbl* gene depletion was directly responsible for the increased basal ERK1/2 phosphorylation levels, we turned to the well-characterized 3T3L1 adipose cell line model. We postulated that if the changes in ERK1/2 signalling were solely due to the depletion of c-CBL in adipose cells and not an indirect or compensatory effect caused by the depletion of c-CBLl expression elsewhere, reduction of c-CBL expression in the 3T3L1 cell line using a shRNA-mediated lentiviral approach would replicate the findings seen in the adipose tissue of the mice. To this end, undifferentiated 3T3L1 cells were either left untreated or infected with lentiviral particles expressing either validated shRNAs for the *Cbl* gene or with lentiviral particles expressing a non-targeting shRNA (NT-shRNA) or with lentiviral particles containing an empty vector as an additional control. Stable cell lines were selected in the presence of puromycin and following amplification, the selected cells were differentiated to obtain fully differentiated adipocytes as we reported previously (Carson *et al*. 2013). Of note, inhibition of *Cbl* by shRNA did not result in changes in the differentiation of 3T3L1 cells into adipocytes (data not shown).

**Figure 5:**
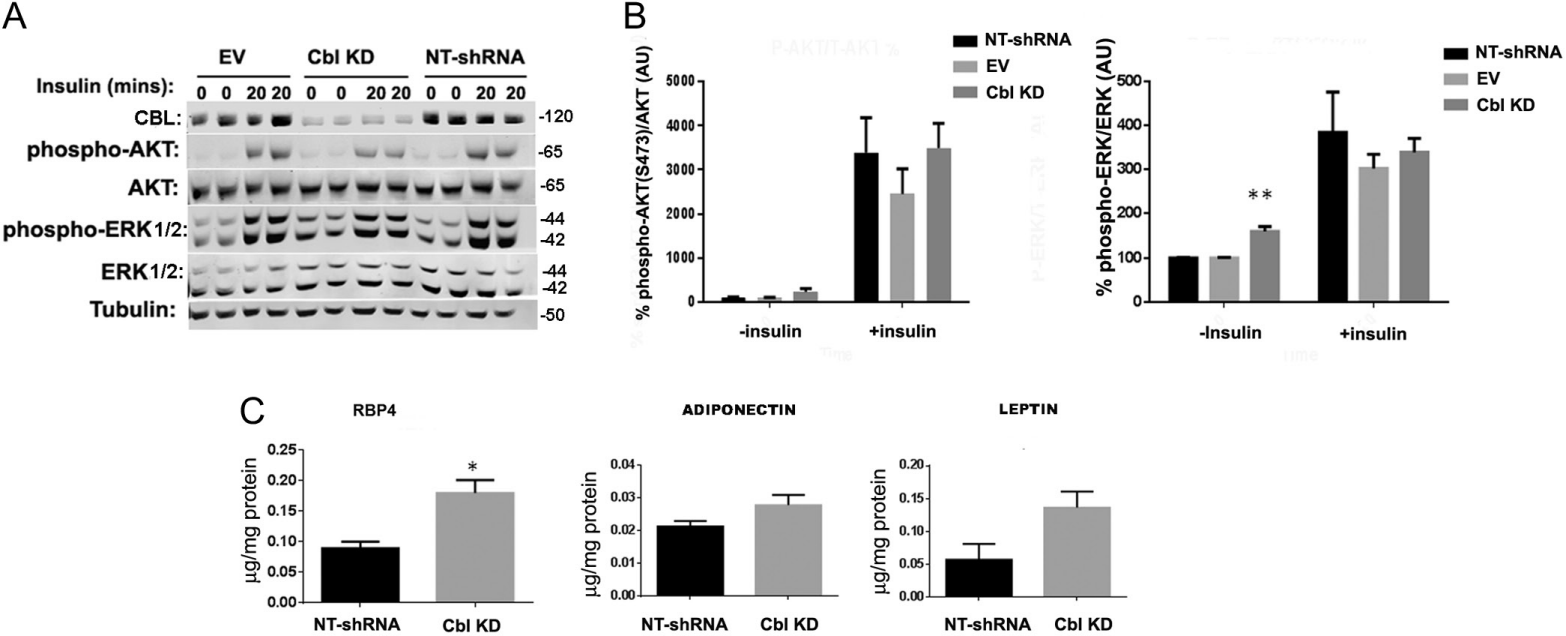
(A) Increased activation of ERK in *Cbl* knockdown 3T3L1 adipocytes. Fully differentiated 3T3L1 adipocyte cells expressing control plasmids (empty vector (EV) or NT-shRNA) or expressing shRNAs for c-*Cbl* (CblKD) were left untreated or stimulated with insulin for 20 min. Cellular lysates were obtained, loaded onto SDS-PAGE and immunoblotted with the indicated antibodies. Representative blot of 5 experiments is shown. (B) shows the quantification in mean ± s.e.m. of intensities in arbitrary units of phospho-AKT (Ser473) and phospho-ERK (T202/204 and Thr185/Tyr187) respectively, obtained in 5 independent experiments. ** indicates *P* < 0.05. (C) Knock down of *Cbl* in 3T3L1 adipocytes increases RBP4 expression. Adipokine expression (RBP4, adiponectin and leptin) were determined by ELISA in whole cellular lysates obtained from control (expressing empty vector (EV) or non-targeting shRNAs) or *Cbl* KD cells (expressing shRNAs for c-*Cbl*). Graphs show the mean ± s.e.m. of *n* = 5 sample replicates. Statistical analysis: One-way ANOVA with Tukey’s test. ** indicates *P* < 0.01.

Expression of c-Cbl in differentiated 3T3L1 cells was significantly reduced by the expression of c-*Cbl* shRNAs compared to control cells expressing an empty vector or a non-targeting shRNA (NT-shRNA) (Fig. 5A). Insulin treatment resulted in the activation of phosphatydilinositol 3-kinase cascade and phosphorylation of AKT in Ser473 to a similar extent in CBL knockdown cells compared to control cells (Fig. 5A and B). However, as detected with the primary adipose tissue of the Cbl^−/−^, we found enhanced basal ERK1/2 phosphorylation levels in the c-Cbl knockdown cells compared to control cells (Fig. 5A and B left panel). Interestingly, concomitantly with these data, we found that RBP4 levels were significantly increased in the c-CBL knockdown cells compared to control cells. Leptin levels were slightly elevated in the CBL-depleted cells albeit this did not reach statistical significance. No changes were observed in adiponectin levels compared to control cells (Fig. 5C).

### Chemical inhibition of ERK1/2 decreases RBP4 expression in adipose tissue explants of CBL-null mice and in CBL-knockdown 3T3L1 adipocytes

**Figure 6:**
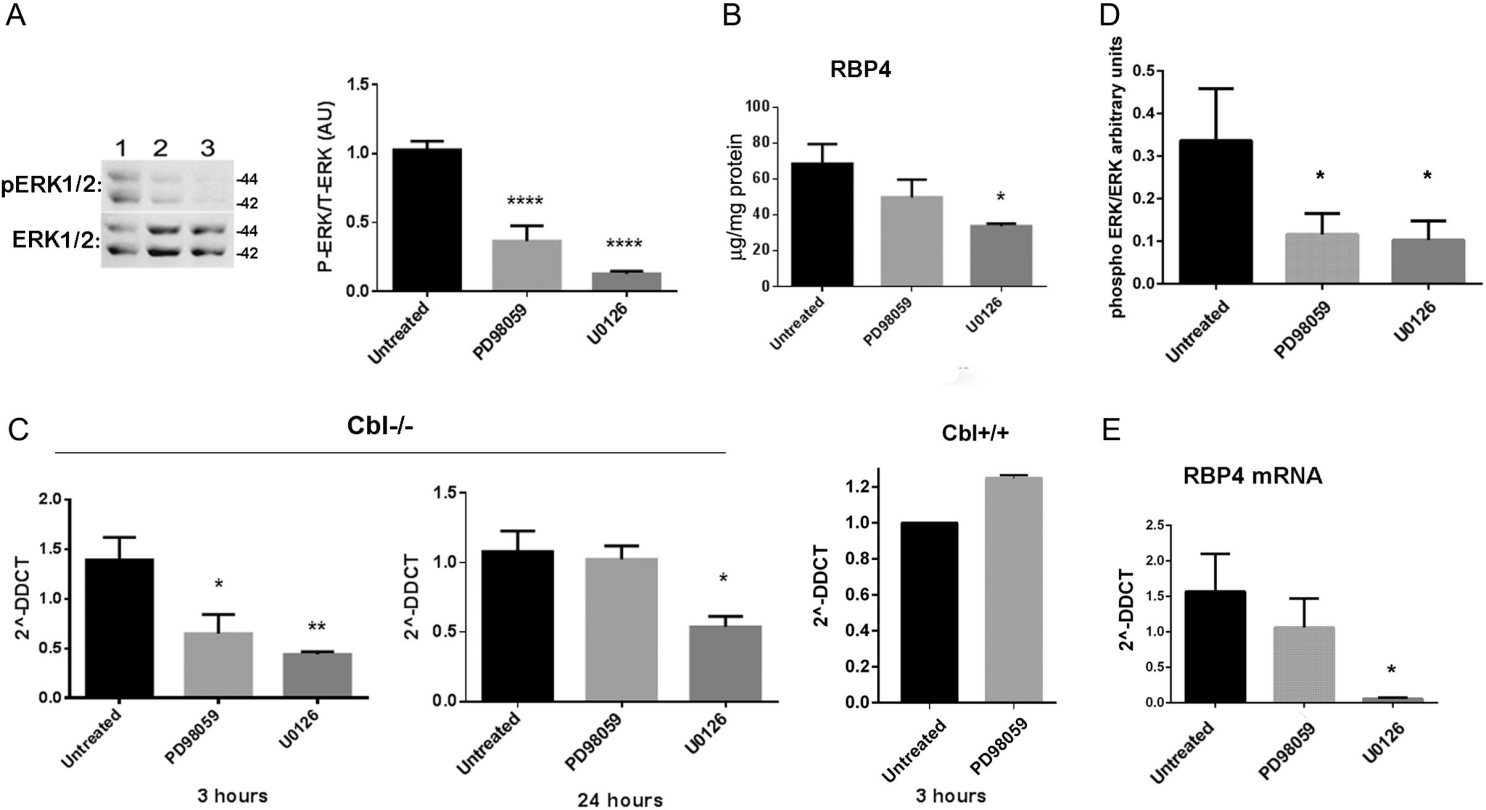
Inhibition of ERK in adipose tissue explants of c-Cbl^−/−^ mice and in Cbl-depleted 3T3L1 adipocytes reduces RBP4 levels. (A) Treatment of adipose tissue explants of CBL-null mice reduces phosphorylation of ERK. Adipose explants were treated with PD98059 or U0126 for 24 h. Tissue lysates were analysed by western blot. Left shows a representative blot, right graph shows quantification of data obtained from *n* = 3 animals with each inhibitor. (B) RBP4 quantification by ELISA in tissue lysates of white adipose tissue explants treated with ERK inhibitors for 24 h. Data are the mean ± s.e.m. from *n* = 3 mice for each inhibitor. (C) mRNA quantification of RBP4 in white adipose tissue explants from Cbl−/− mice left untreated or treated with ERK inhibitors for 3 h or 24 h. Graphs are mean ± s.e.m. of relative quantification to 18S as housekeeping gene, data obtained from *n* = 2 mice for each inhibitor, each sample quantified in triplicate. Right panel: mRNA quantification of RBP4 in white adipose tissue explants from Cbl+/+ mice incubated for 3 h with PD98059. (D) *Cbl* KD 3T3L1 adipocytes were left untreated or treated with ERK inhibitors for 15 h. Cell lysates were obtained and analysed by Western blot. Graph is quantitation of an experiment done in triplicate cell dishes. Representative of two independent experiments done in triplicate. (E) mRNA levels of *Rbp4* were quantitated in *Cbl-depleted* 3T3L1 adipocytes left untreated or incubated with inhibitors for 15 h. Graph shows mean and s.e.m. Representative experiment of *n* = 2, with 3 biological replicates. Statistical analysis: one-way ANOVA vs untreated with Sidak’s *post hoc* test. * indicates *P* < 0.05, **P < 0.01; ****P < 0.0001.

Based on the earlier data, we postulated that the increase in ERK1/2 activation seen in adipocytes depleted of CBL1 may be responsible for the elevated expression of RPB4. To test this hypothesis, we examined the effects of chemical inhibition of ERK1/2 in the adipose tissue explants of the *Cbl*-null mice and in 3T3L1 *Cbl* KD adipocytes. First, visceral adipose tissue explants were obtained and cultured *in vitro* in DMEM media in the absence or presence of two chemical inhibitors (10 µM of PD98059 and 20 µM U0126) of MEK1/2, the upstream kinases that regulate ERK1/2. Subsequently, adipose tissue lysates were obtained and immunoblotted with antibodies for phospho-ERK1/2 to determine the level of inhibition. Both inhibitors significantly decreased ERK1/2 phosphorylation after 24-h treatment although U0126 seemed to achieve greater inhibition (Fig. 6A). We then determined the effect of this inhibition on RBP4 expression. Concomitantly with the reduction in ERK1/2 activation the content of RBP4 protein in the tissue was reduced with U0126 treatment (Fig. 6B). At the mRNA level, treatment with both inhibitors reduced *Rbp4* mRNA levels maximally after 3 h of treatment (Fig. 6C). *Rbp4* mRNA levels recovered completely after 24 h following the treatment with PD98059, whereas a 50% decrease was still noted for U0126 at this time point (Fig. 6C). As expected, the inhibitor PD98059 did not reduce the expression of *Rbp4* in adipose tissue of WT mice (Fig. 6C).

We confirmed these data in the 3T3L1 adipocytes cell line and found that chemical inhibition of ERK1/2 activation in these cells (Fig. 6D) also reduced the levels of mRNA for *Rbp4* (Fig. 6E).

### Oestrogen receptor regulates RBP4 expression in adipocytes

**Figure 7:**
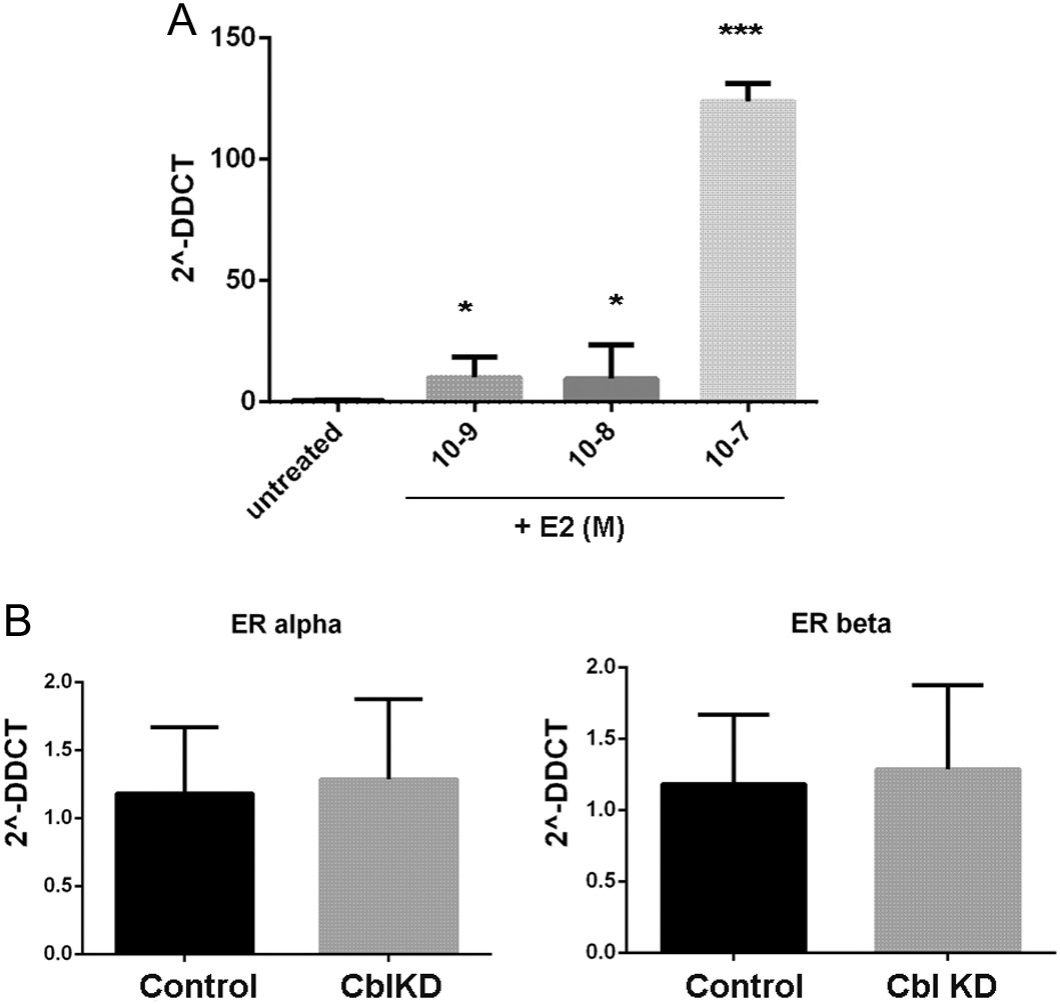
(A) 17β-estradiol increases *Rbp4* mRNA in 3T3l1 cells. Differentiated adipocytes were treated with E2 at a range of concentrations from 0 to 10^−7^ M as indicated for 15 h. Total RNA was isolated and *Rbp4* mRNA levels analysed by real-time PCR as described in the methods. Graphs shows mean and s.e.m. of *n* = 3 cell dishes. One-Way ANOVA *** indicates *P* < 0.01. (B) Expression of ER receptors in Cbl KD 3T3L1 cells. Total RNA was isolated from control or *Cbl-*depleted 3T3L1 adipocytes and the mRNA levels of ERα and ERβ were determined by qPCR as described in ‘Methods’ section. Graphs shows mean ± s.e.m. of *n* = 3 cell dishes. Statistical analysis: One-way ANOVA, non-significant.

Since our findings on RBP4 expression in adipose tissue were confined to female mice and WAT expresses abundant ER receptors, we postulated that 17β-estradiol may be involved in regulating RBP4. To assess this, we first used UCSC Genome Browser (http://genome.UCSC.edu) to examine the human and mouse gene sequences. We found oestrogen receptor alpha-responsive elements (ERE) in the human promoter, in an area that is highly conserved to the mouse gene (data not shown). This suggested that oestrogens may contribute to RBP4 gene regulation. To determine whether 17β-estradiol regulates *Rbp4* expression in adipocytes, we treated differentiated 3T3L1 adipocytes with increasing concentrations (0, 1, 10 and 100 nM) of 17β-estradiol for 15 h. Total RNA was subsequently harvested and mRNA levels of *Rbp4* were determined by qPCR. We found that 17β-estradiol increased *Rbp4* mRNA levels in adipocytes (Fig. 7A).

### Cbl depletion increases the phosphorylation of estrogen receptor alpha

Next, we questioned whether *Cbl* depletion in adipocytes would increase the expression or activation of ER receptor. We determined the expression of *Era* and *Erb* by qPCR in control cells and in cells stably expressing shRNAs *Cbl* KD. We found that the mRNA levels for ER receptors in *Cbl*-depleted cells were similar to those observed in control cells (Fig. 7B).

**Figure 8:**
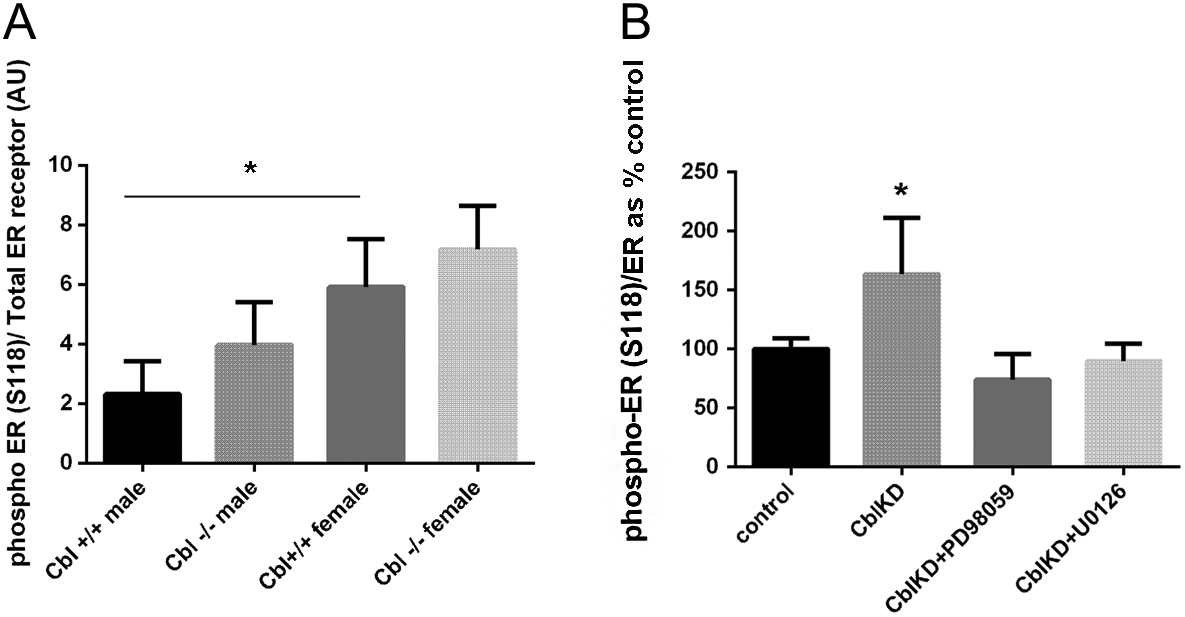
Higher levels of ERa S118 phosphorylation in *Cbl*-depleted adipocytes. (A) White adipose tissue extracts obtained from c-Cbl^−/−^ or c-Cbl^+/+^ mice (*n* = 3 male and *n* = 3 female for each genotype) were immunoblotted with anti-phospho ERα (S118) antibody or ER antibody as loading control. The blot was quantified with Image J. Graph shows mean ± s.e.m. of intensities in arbitrary units. Statistical analysis: ANOVA with Sidak’s multiple comparison. * indicates *P* < 0.05. (B) Whole cell lysates obtained from either control 3T3L1 adipocytes or *Cbl*-depleted cells were immunoblotted with with anti-phospho ERa (S118) antibody or total ER as loading control. Graph shows a the quantification in Image J of *n* = 3–5 replicate samples per group and represents the mean ± s.e.m. of phospho ERa/ER ratio in arbitrary units as % of control cells, which do not express shRNAs for *Cbl.* Statistical analysis: ANOVA with Dunnett’s test. * indicates *P* < 0.05 vs control.

Existing literature demonstrates that ERa can be activated by phosphorylation at Ser118, a consensus phosphorylation site for ERK1/2. We next tested whether *Cbl* depletion could result in increased phosphorylation of ERa. To this end, we immunoblotted WAT extracts obtained from c-Cbl^−/−^ and c-Cbl^+/+^ mice and 3T3L1 lysates obtained from control or CBL knockdown cells, with a phosphoERa S118-specific antibody and a total ERa antibody as loading control. We found that the phospho ER (S118) levels normalized to total ER receptor were slightly elevated in the CBL-null mice, albeit it did not reach statistical significance (Fig. 8A). In 3T3L1 adipocytes, CBL-depleted cells showed increased ER S118 phosphorylation, and as expected, this decreased dramatically upon exposure to MEK1 and MEK2 inhibitors PD98059 and U0126 (Fig. 8B).

## Discussion

CBL-null mice made in the C57BL6 background did not display any significant growth differences compared with the WT mice. The male mice replicated the insulin sensitivity phenotype described previously on the Jvs129 background (Molero *et al*. 2004, 2006), whereas females were not significantly different than WT. It was reported that CBL depletion increases muscle metabolism (Molero *et al*. 2004) enhanced energy expenditure and reduced adiposity. The c-Cbl-null mice used in our studies did not show any adipose tissue morphology abnormalities or differences adipocyte cell size. These findings suggest small differences in the phenotype that may be attributable to distinct genomic background.

However, we detected some differences in the adipokine content of the WAT of female c-CBL-null mice. Content of adiponectin was decreased while leptin and RBP4 were increased in the female Cbl^−/−^ compared to Cbl^+/+^ mice. Reduced adiponectin levels and increased RBP4 plasma levels have been shown to correlate with states of insulin resistance such as obesity in both animal and humans (Yang *et al*. 2005, Tan *et al*. 2007). The increase in RBP4 content was relatively small and only detected in WAT with no changes seen in the liver or in the circulating plasma levels. Since the liver is the main producer of RBP4 protein contributing to 80% of the plasma levels, that explains the limited impact on circulating RBP4 and in overall insulin sensitivity. However, local changes of RBP4 levels in WAT are important in determining a local inflammatory response (Moraes-Vieira *et al*. 2016). Norseen and coworkers (Norseen *et al*. 2012) have recently reported that transgenic overexpression of RBP4 in adipose tissue increases adipose tissue inflammation through the activation of macrophages via JNK, TLR4 and NFκB signalling pathways. We did not detect any local increase in the abundance of proinflammatory cytokines IL6 or TNFa in WAT, which suggests RBP4 levels in the CBL-null mice did not reach the threshold necessary to increase local macrophage inflammation response.

We found increased basal activation of ERK1/2 in the adipose tissue of c-Cbl^−/−^ mice and in 3T3L1 adipocyte cells depleted of c-CBL. This correlated with the increase in RBP4 and leptin expression in adipose tissue. We did not detect any increase in the expression of RBP4 or raised basal ERK1/2 phosphorylation in the liver of c-Cbl^−/−^ mice (data not shown). Furthermore, chemical inhibition of ERK pathway in c-CBL-depleted adipocytes decreased RBP4 levels. These findings suggest that c-CBL may regulate RBP4 expression through ERK1/2 activation. Indeed, c-CBL proteins have dual functionalities in cells. They act as adaptor proteins for tyrosine kinase receptors, including the insulin receptor and recruit signalling proteins in the caveolae of adipose cells (Watson & Pessin 2001). In addition, c-CBL proteins can function as an E3-ubiquitin ligase enzyme, thus facilitating the degradation of proteins. Molero and coworkers (Molero *et al*. 2004) had found increased insulin receptor expression in the skeletal muscle of Cbl^−/−^ mice. However, we did not detect any changes in the expression levels of the glucose transporter GLUT4 or IRS1. Furthermore, signalling through PI 3-kinase in response to insulin was unremarkable compared to the WT mice indicating equal insulin receptor substrate 1 and PI 3-kinase activation. It remains possible, however, that the effects seen on increased ERK1/2 activation may be dependent on CBL action as an E3-ubiquitin ligase on ERK1/2 regulatory proteins, for example, targeting an upstream ERK kinase or through an inhibitory effect of an ERK phosphatase. Further research is necessary to identify the molecular mechanisms for increased basal ERK1/2 phosphorylation in c-CBL-depleted adipose cells.

There was a clear gender dimorphism effect observed with RBP4 seen elevated only in female mice. Similar findings were found previously in humans (Kos *et al*. 2011). Both ERa and ERb oestrogen receptors are present in WAT with ERa being the most abundant. These receptors play an important role during adipose tissue differentiation. Binding of oestradiol to the ERa facilitates ligand-dependent activation and transactivation of oestrogen response elements (ERE) in target genes, which activate or repress gene expression. The human *Rbp4* promoter has ERE sequences in conserved regions, which suggest a role for ER in the expression of RBP4, in support of this, we found that incubation of mouse adipose cells with 17β-estradiol increased *Rbp4* mRNA levels. Our data are consistent with previous findings by Jung and coworkers (Jung *et al*. 2013) who found that in 3T3L1 adipocytes, ERa but not ERb activation increased *Rbp4* mRNA levels.

We did not determine the oestrous cycle in our female mice or indeed measured the circulating estradiol levels to correlate them with *Rbp4* expression levels. However, the existing literature in human studies has shown a correlation of RBP4 serum levels with oestradiol and oestradiol/testosterone levels (Mohasseb & Khalil 2014), which supports the role of ER in the *in vivo* regulation of *Rbp4* expression. The effects of other female hormones released during the oestrous cycle on the expression of *Rbp4*, however, have not been extensively studied. A recent study in heifers showed Rbp4 mRNA and protein levels raised in the endometrium during the dioestrus phase with elevated progesterone levels (Mullen *et al*. 2012). This may be related to the role of retinoic acid signalling in the expression of proteins important for embryo implantation (Ma *et al*. 2012). Unfortunately, the authors did not measure *Rbp4* levels in WAT of these animals.

The ER receptors are members of the nuclear hormone receptor superfamily of transcription factors that bind ERE sequences as homo or heterodimers. ERs contain two transcription activation functions: AF1 located in the N-terminal A/B domain and the AF2 located in the C terminal domain. AF2 is activated through ligand (hormone) binding, whereas AF1 can modulate gene transcription in the absence of ligand (Murphy *et al*. 2011), but this is weak.

ER activity can be modulated by intracellular signalling pathways (Barone *et al*. 2010) that phosphorylate ERa. AKT and ERK1/2 phosphorylation sites on ERa map at S167 for AKT and S118 for ERK1/2 both within the AF1 domain. While phosphorylation at these sites leads to ligand-independent activation of ERa (Murphy *et al*. 2011), AF1 works to synergize with AF2 in the promotion of ligand-dependent transcription activation by the receptor (reviewed by Tsai & O’Malley 1994). Thus, we postulate that this mechanism may operate in the female CBL-null mice to enhance the expression of *Rbp4*. Based on our findings, we propose that along with the increased number of ER receptors and oestrogen circulating levels present in females, c-CBL depletion may potentiate ER activity through ERK1/2-mediated phosphorylation of ER at S118, which results in higher RBP4 expression.

All our data suggest that inhibition of *Cbl* in adipose tissue will increase RBP4 expression locally. We previously reported that CBL signalling is impaired in animal models of insulin deficiency and in obesity (Gupte & Mora 2006). While our findings need to be explored in the context of human adipose tissue, this study reveals a potential new molecular mechanism that may contribute locally to the dysregulation of RBP4 that occurs in obesity and insulin resistance.

## Declaration of interest

The authors declare that there is no conflict of interest that could be perceived as prejudicing the impartiality of the research reported.

## Funding

G I A was a recipient of a PhD Studentship from the Kurdistan Government of Iraq.

## References

[bib1] Aleffi S Petrai I Bertolani C Parola M Colombatto S Novo E Vizzutti F Anania F Milani S Rombouts K 2005 Upregulation of proinflammatory and proangiogenic cytokines by leptin in human hepatic stellate cells. Hepatology 42 1339–1348. (10.1002/hep.20965) 16317688

[bib2] Arita Y Kihara S Ouchi N Takahashi M Maeda K Miyagawa J Hotta K Shimomura I Nakamura T Miyaoka K 1999 Paradoxical decrease of an adipose-specific protein, adiponectin, in obesity. Biochemical and Biophysical Research Communications 257 79–83. (10.1006/bbrc.1999.0255) 10092513

[bib3] Barone I Bursco L Fuqua S 2010 Estrogen receptor mutations and chances in downstream gene expression and signalling. Clinical Cancer Research 16 2702–2708. (10.1158/1078-0432.CCR-09-1753) 20427689 PMC4477803

[bib4] Baumann C Ribon V Kanzaki M Thurmond D Mora S Shigematsu S Bickel P Pessin J Saltiel A 2000 CAP defines a second signalling pathway required for insulin-stimulated glucose transport. Nature 407 202–207. (10.1038/35025089) 11001060

[bib5] Berg A Combs T Scherer P 2002 ACRP30/adiponectin: an adipokine regulating glucose and lipid metabolism. Endocrinology and Metabolism 13 84–89. 10.1016/s1043-2760(01)00524-0 11854024

[bib6] Bouatia-Naji N Meyre D Lobbens S Seron K Fumeron F Balkau B Heude B Jouret B Scherer PE Dina C 2006 ACDC/adiponectin polymorphisms are associated with severe childhood and adult obesity. Diabetes 55 545–550. (10.2337/diabetes.55.02.06.db05-0971) 16443793

[bib7] Boyraz M Cekmez F Karaoglu A Cinaz P Durak M Bideci A 2013 Relationship of adipokines (adiponectin, resistin and RBP4) with metabolic syndrome components in pubertal obese children. Biomarkers in Medicine 7 423–428. (10.2217/bmm.13.14) 23734806

[bib8] Carson B Del Bas J Moreno-Navarrete J Fernandez-Real J Mora S 2013 The rab11 effector FIP1 regulates adiponectin trafficking and secretion. PLoS ONE 8 e74687 (10.1371/journal.pone.0074687) 24040321 PMC3770573

[bib9] Chang L Adams RD Saltiel AR 2002 The TC10-interacting protein CIP4/2 is required for insulin-stimulated Glut4 translocation in 3T3L1 adipocytes. PNAS 99 12835–12840. (10.1073/pnas.202495599) 12242347 PMC130546

[bib10] Chiang S Baumann C Kanzaki M Thurmond D Watson R Neudauer C Macara I Pessin J Saltiel A 2001 Insulin-stimulated GLUT4 translocation requires the CAP-dependent activation of TC10. Nature 410 944–948. (10.1038/35073608) 11309621

[bib11] Fried SK Moustaid-Moussa N 2001 Culture of adipose tissue and isolated adipocytes. Methods in Molecular Biology 155 197–212. 11293072 10.1385/1-59259-231-7:197

[bib12] Friedman J Halaas J 1998 Leptin and the regulation of body weight in mammals. Nature 395 763–770. (10.1038/27376) 9796811

[bib13] Gainsford T Willson TA Metcalf D Handman E McFarlane C Ng A Nicola N Alexander W Hilton D 1996 Leptin can induce proliferation, differentiation, and functional activation of hemopoietic cells. PNAS 93 14564–14568. (10.1073/pnas.93.25.14564) 8962092 PMC26173

[bib14] Graham TE Yang Q Bluher M Hammarstedt A. C-r T.P. Henry RR Wason C Oberbach A Jansson P Smith U 2006 Retinol binding protein 4 and insulin resistance in lean, obese and diabetic subjects. New England Journal of Medicine 354 2552–2563. (10.1056/NEJMoa054862) 16775236

[bib15] Gupte A Mora S 2006 Activation of the Cbl insulin signaling pathway in cardiac muscle; dysregulation in obesity and diabetes. Biochemical and Biophysical Research Communications 342 751–757. (10.1016/j.bbrc.2006.02.023) 16494846

[bib16] Jung US Jeong K Kang J Yi KW Shin J Seo H Kim T Kim S Hur J 2013 Effects of estrogen receptor alpha and beta on the expression of visfatin and retinol binding protein 4 in 3T3L1 adipocytes. International Journal of Molecular Medicine 32 723–728. (10.3892/ijmm.2013.1440) 23857051

[bib17] Kadowaki T Yamauchi T Kubota N Hara K Ueki K Tobe K 2006 Adiponectin and adiponectin receptors in insulin resistance, diabetes, and the metabolic syndrome. Journal of Clinical Investigation 116 1784–1792. (10.1172/JCI29126) 16823476 PMC1483172

[bib18] Klöting N Graham T Berndt J Kralisch S Kovacs P Wason C Fasshauer M Schön M Stumvoll M Blüher M 2007 Serum retinol-binding protein is more highly expressed in visceral than in subcutaneous adipose tissue and is a marker of intra-abdominal fat mass. Cell Metabolism 6 79–87. 17618858 10.1016/j.cmet.2007.06.002

[bib19] Kos K Wong S Tan BK Kerrigan D Randeva HS Pinkney JH Wilding JP 2011 Human RBP4 adipose tissue expression is gender specific and influenced by leptin. Clinical Endocrinology 74 197–205. (10.1111/j.1365-2265.2010.03892.x) 21039728

[bib20] Kovacs P Geyer M Berndt J Kloting N Graham TE Bottcher Y Enigk B Tonjes A Schleinitz D Schon MR 2007 Effects of genetic variation in the human retinol binding protein-4 gene (RBP4) on insulin resistance and fat depot-specific mRNA expression. Diabetes 56 3095–3100. (10.2337/db06-1647) 17728376

[bib21] Kwon H Pessin J 2013 Adipokines mediate inflammation and insulin resistance. Frontiers in Endocrinology 4 71. 23781214 10.3389/fendo.2013.00071 PMC3679475

[bib22] Lee SA Yuen JJ Jiang H Kahn BB Blaner WS 2016 Adipocyte-specific overexpression of retinol-binding protein 4 causes hepatic steatosis in mice. Hepatology 64 1534–1546. (10.1002/hep.28659) 27227735 PMC5074895

[bib23] Liu J Kimura A Baumann C Saltiel A 2002 APS facilitates c-Cbl tyrosine phosphorylation and GLUT4 translocation in response to insulin in 3T3-L1 adipocytes. Molecular and Cellular Biology 11 3599–3609. (10.1128/MCB.22.11.3599-3609.2002) PMC133825 11997497

[bib24] Liu J DeYoung S Hwang J O’Leary E Saltiel A 2003 The roles of Cbl-b and c-Cbl in insulin-stimulated glucose transport. Journal of Biological Chemistry 278 36754–36762. (10.1074/jbc.M300664200) 12842890

[bib25] Lovren F Pan Y Quan A Szmitko P Singh K Shukla P Gupta M Chan L Al-Omran M Teoh H 2010 Adiponectin primes human monocytes into alternative anti-inflammatory M2 macrophages. American Journal of Physiology: Heart and Circulatory Physiology 299 H656–H663. (10.1152/ajpheart.00115.2010) 20622108 PMC2944489

[bib26] Ma J Han B Peng J 2012 Retinoic acid synthesis and metabolism are concurrent in the mouse uterus during peri-implantation. Cell and Tissue Research 350 525–537. (10.1007/s00441-012-1507-4) 23053054

[bib27] Mandal P Pratt B Barnes MM McMullen M Nagy L 2011 Molecular mechanism for adiponectin-dependent M2 macrophage polarization:link between the metabolic and innate immune activity of full-length adiponectin. Journal of Biological Chemistry 286 13460–13469. (10.1074/jbc.M110.204644) 21357416 PMC3075692

[bib28] Mohasseb M Khalil GI 2014 Estradiol testosterone ration, serum retinol binding protein 4 and insulin resistance in overweight and obese Egyptian men. Journal of Research in Obesity 2014 article ID 837473 (10.5171/2014.837473)

[bib29] Molero JC Jensen TE Withers PC Couzens M Herzog H Thien CB Langdon WY Walder K Murphy MA Bowtell DD 2004 c-Cbl-deficient mice have reduced adiposity, higher energy expenditure, and improved peripheral insulin action. Journal of Clinical Investigation 114 1326–1333. (10.1172/JCI21480) 15520865 PMC524227

[bib30] Molero JC Turner N Thien CB Langdon WY James DE Cooney GJ 2006 Genetic ablation of the c-Cbl ubiquitin ligase domain results in increased energy expenditure and improved insulin action. Diabetes 55 3411–3417. (10.2337/db06-0955) 17130487

[bib31] Mora S Yang C Ryder JW Boeglin D Pessin JE 2001 The MEF2A and MEF2D isoforms are differentially regulated in muscle and adipose tissue during states of insulin deficiency. Endocrinology 142 1999–2004. (10.1210/endo.142.5.8160) 11316766

[bib32] Moraes-Vieira PM Yore MM Dwyer PM Syed I Aryal P Kahn BB 2014 RBP4 activates antigen-presenting cells, leading to adipose tissue inflammation and systemic insulin resistance. Cell Metabolism 19 512–526. (10.1016/j.cmet.2014.01.018) 24606904 PMC4078000

[bib33] Moraes-Vieira PM Castoldi A Aryal P Wellenstein K Peroni OD Kahn BB 2016 Antigen presentation and T-cell activation are critical for RBP4-induced insulin resistance. Diabetes 65 1317–1327. (10.2337/db15-1696) 26936962 PMC4839203

[bib34] Mullen M Forde N Diskin M Nally J Crowe M 2012 Alterations in systemic concentrations of progesterone during the early luteal phase affect RBP4 expression in the bovine uterus. Reproduction, Fertility, and Development 24 715–722. (10.1071/RD11246) 22697121

[bib35] Murphy LC Seekallu SV Watson PH 2011 Clinical significance of estrogen receptor phosphorylation. Endocrine-Related Cancer 18 R1–R14. (10.1677/ERC-10-0070) 21149515

[bib36] Norseen J Hosooka T Hammarstedt A Yore M Kant S Aryal P Kiernan UA Phillips DA Maruyama H Kraus BJ 2012 Retinol-binding protein 4 inhibits insulin signaling in adipocytes by inducing proinflammatory cytokines in macrophages through a c-Jun N-terminal kinase- and toll-like receptor 4-dependent and retinol-independent mechanism. Molecular and Cellular Biology 32 2010–2019. (10.1128/MCB.06193-11) 22431523 PMC3347417

[bib37] Ohashi K Parker JL Ouchi N Higuchi A Vita JA Gokce N Pedersen AA Kalthoff C Tullin S Sams A 2010 Adiponectin promotes macrophage polarization toward an anti-inflammatory phenotype. Journal of Biological Chemistry 285 6153–6160. (10.1074/jbc.M109.088708) 20028977 PMC2825410

[bib38] Ohashi K Shibata R Murohara T Ouchi N 2014 Role of anti inflammatory adipokines in obesity related diseases. Trends in Endocrinology and Metabolism 25 348–355. (10.1016/j.tem.2014.03.009) 24746980

[bib39] Ohashi K Yuasa D Shibata R Murohara T Ouchi N 2015 Adiponectin as a target in obesity-related inflammatory state. Endocrine, Metabolic and Immune Disorders: Drug Targets 15 145–150. (10.2174/1871530315666150316122709) 25772181

[bib40] Okamoto Y Kihara S Ouchi N Nishida M Arita Y Kumada M Ohashi K Sakai N Shimomura I Kobayashi H 2002 Adiponectin reduces atherosclerosis in apolipoprotein E-deficient mice. Circulation 106 2767–2770. (10.1161/01.CIR.0000042707.50032.19) 12451000

[bib41] Okamoto Y Folco EJ Minami M Wara AK Feinberg MW Sukhova GK Colvin RA Kihara S Funahashi T Luster AD 2008 Adiponectin inhibits the production of CXC receptor 3 chemokine ligands in macrophages and reduces T-lymphocyte recruitment in atherogenesis. Circulation Research 102 218–225. (10.1161/CIRCRESAHA.107.164988) 17991878

[bib42] Ouchi N Kihara S Arita Y Nishida M Matsuyama A Okamoto Y Ishigami M Kuriyama H Kishida K Nishizawa H 2001 Adipocyte-derived plasma protein, adiponectin, suppresses lipid accumulation and class A scavenger receptor expression in human monocyte-derived macrophages. Circulation 103 1057–1063. (10.1161/01.CIR.103.8.1057) 11222466

[bib43] Ouchi N Parker J Lugus J Walsh K 2011 Adipokines in inflammation and metabolic disease. Nature Reviews Immunology 11 85–97. (10.1038/nri2921) PMC3518031 21252989

[bib44] Pajvani UB Scherer PE 2003 Adiponectin: systemic contributor to insulin sensitivity. Current Diabetes Reports 3 207–213. (10.1007/s11892-003-0065-2) 12762967

[bib45] Takemura Y Ouchi N Shibata R Aprahamian T Kirber MT Summer RS Kihara S Walsh K 2007 Adiponectin modulates inflammatory reactions via calreticulin receptor-dependent clearance of early apoptotic bodies. Journal of Clinical Investigation 117 375–386. (10.1172/JCI29709) 17256056 PMC1770947

[bib46] Tamori Y Sakaue H Kasuga M 2006 RBP4, an unexpected adipokine. Nature Medicine 12 30–31; discussion 31. (10.1038/nm0106-30) 16397554

[bib47] Tan BK Chen J Lehnert H Kennedy R Randeva HS 2007 Raised serum, adipocyte, and adipose tissue retinol-binding protein 4 in overweight women with polycystic ovary syndrome: effects of gonadal and adrenal steroids. Journal of Clinical Endocrinology and Metabolism 92 2764–2772. (10.1210/jc.2007-0091) 17456573

[bib48] Tsai M O’Malley B 1994 Modular mechanisms of action of steroid/tyroid receptor superfamily members. Annual Review of Biochemistry 63 451–486. (10.1146/annurev.bi.63.070194.002315) 7979245

[bib49] Watson R Pessin J 2001 Transmembrane domain length determines intracellular membrane compartment localization of syntaxins 3, 4 and 5. American Journal of Physiology: Cell Physiology 281 C215–C223. 11401844 10.1152/ajpcell.2001.281.1.C215

[bib50] Xie L Boyle D Sanford D Scherer P Pessin J Mora S 2006 Intracellular trafficking and secretion of adiponectin is dependent on GGA coated vesicles. Journal of Biological Chemistry 281 7253–7259. (10.1074/jbc.M511313200) 16407204

[bib51] Xie L O’Reilly CP Chapes SK Mora S 2008 Adiponectin and leptin are secreted through distinct trafficking pathways in adipocytes. Biochimica et Biophysica Acta 1782 99–108. (10.1016/j.bbadis.2007.12.003) 18179777 PMC2292133

[bib52] Yamanuchi T Kamon J Minokoshi Y Ito Y Waki H Uchida S Yamashita S Noda M Kita S Eto K 2002 Adiponectin stimulates glucose utilization and fatty acid activating AMP-activated protein kinase. Nature Medicine 8 1288–1295. (10.1038/nm788) 12368907

[bib53] Yang C Mora S Ryder JW Coker KJ Hansen P Allen LA Pessin JE 2001 VAMP3 null mice display normal constitutive, insulin- and exercise-regulated vesicle trafficking. Molecular and Cellular Biology 21 1573–1580. (10.1128/MCB.21.5.1573-1580.2001) 11238894 PMC86703

[bib54] Yang Q Graham TE Mody N Preitner F Peroni OD Zabolotny JM Kotani K quadro L Khan B 2005 Serum retinol binding protein 4 contributes to insuin resistance in obesity and type 2 diabetes. Nature 436 356–362. (10.1038/nature03711) 16034410

[bib55] Yang C Aye C Li X Diaz-Ramos A Zorzano A Mora S 2012 Mitochondrial dysfunction in insulin resistance: differential contributions of chronic insulin and saturated fatty acid exposure in muscle cells. Bioscience Reports 32 465–478. (10.1042/BSR20120034) 22742515 PMC3475448

[bib56] Yao-Borengasser A Varma V Bodles A Rasouli N Phanavanh B Lee M Starks T Kern L Spencer H Rashidi A 2007 Retinol binding protein 4 expression in humans: relationship to insulin resistance, inflammation, and response to pioglitazone. Journal of Clinical Endocrinology and Metabolism 92 2590–2597. (10.1210/jc.2006-0816) 17595259 PMC2893415

[bib57] Zhang Y Proenca R Maffei M Barone M Leopold L Friedman J 1994 Positional cloning of the mouse obese gene and its human homologue. Nature 372 425–432. (10.1038/372425a0) 7984236

